# Severe polymicrobial and fungal periprosthetic osteomyelitis persisting after hip disarticulations treated with caspofungin in risk patients: a case series

**DOI:** 10.1186/s12941-021-00490-3

**Published:** 2021-12-31

**Authors:** Andreas Enz, Silke Müller, Wolfram Mittelmeier, Annett Klinder

**Affiliations:** 1 Orthopedic Clinic and Policlinic, University Medical Center Rostock, Doberaner Str. 142, 18057 Rostock, Germany; 2 Institute of Pharmacology and Toxicology, University medical center Rostock, Schillingallee 70, 18057 Rostock, Germany

**Keywords:** Echinocandin, Periprosthetic joint infection, Fungi, Osteomyelitis, Mixed infections

## Abstract

**Background:**

Periprosthetic fungal infections are considered rare and opportunistic infections. Treatment is difficult, and established standards do not yet exist. The choice of the appropriate antifungal drug might affect the patient outcome.

**Cases:**

All the three cases presented showed polybacterial recurrent infection of the revision hip arthroplasty. All patients were of younger age, had multiple revisions of the endoprosthesis, each had a large partial femoral replacement greater than 40% of the femoral length, gentamycin-loaded cement, and a long anchoring distance of the used intramedullary stem. Due to the severe life-threatening infection with deep osteomyelitis, an amputation had to be performed. However, despite surgical intervention, the fungal dominated infection persisted. Finally, only the use of caspofungin allowed permanent infection control.

**Conclusion:**

The polybacterial infection is driven by the symbiosis between fungi and bacteria. Therefore, eradication of the fungus is required to achieve elimination of the bacteria. Antimycotics of the echinocandin-class, such as caspofungin, may be considered as initial treatment.

## Background

Treatment of endoprosthesis infection is a major challenge in the field of endoprosthetics and one of the most serious complications. Various standard procedures for the treatment of implant-associated infections exist [1, 2], but polymicrobial infections and especially mixed infections of fungi and bacteria are highly detrimental for patients and demanding for the surgical team, often requiring an interdisciplinary setup. Fungal infections are described in the literature as rare, underestimated and difficult to detect [3]. Frequently, there is a delay in the detection of the fungus. There are only limited recommendations in the literature for the treatment of periprosthetic infections with fungi and polymicrobial pathogens [4, 5]. In addition to surgical treatment, pharmacotherapy is an important part of the treatment [6, 7]. In particular, the biofilm-forming properties of *Candida* and the symbiosis with bacteria like *Staphylococcus aureus* may prevent successful treatment of the infection [8]. This makes the choice and use of a biofilm-cracking antifungal agent very important for successful treatment of the infection [9]. With the report of these three cases, we would like to highlight the use of caspofungin and the necessity of a radical surgical therapy to save the patient's life.

## Case presentations

The following case presentations were selected to illustrate representative amputation cases with fungal infections and the treatment efforts to control the periprosthetic joint infection (PJI). Consecutive changes in the patients' bacterial and fungal spectrum during hospitalisation after the surgical disarticulation and subsequent surgeries are shown in Table 1. In all cases, the fungal infection was in the central area of the acetabular hip region.

**Table 1:** List of detected pathogens including their antibiotic susceptibility profile during the reported hospital stay

Number of consecutive microbiological testings	Pathogens detected during reported hospital stay involving disarticulation [resistant to]		
	Case 1	Case 2	Case 3
1	*E. faecalis* [G, S, Te, Co, E] *S. epidermidis* (MRSE) [O, Am, Cf, I, Le, M, G, Te, Co, E, Cl, Fo, R]	*E. faecalis* [G, Te, Co, E]	Negative
2	*E. faecalis* [G, S, Te, Co, E] *S. aureus* (MRSA) [O, Am, Cf, I, Le, M, E, Cl]	*E. faecalis* [G, Co] *S. epidermidis* (MRSE) [O, Am, Cf, I, Le, G, Te, E, Cl, Fu]	*P. mirabilis* [Tg] *E. faecalis* [Co]
3	*E. faecalis* [G, S, Te, Co, E] *S. aureus* (MRSA) [O, Am, Cf, I, Le, M, E, Cl]	*E. faecalis* [G, Co] *S. epidermidis* [O, Am, Cf, I, Le, G, Te, E, Cl, Fu, Li]	*P. mirabilis* [Tg] *E. faecalis* [Te, Co, E]
4	*E. faecalis* [G, S, Te, Co, E] *S. aureus* (MRSA) [O, Am, Cf, I, Le, M, E, Cl]	*E. faecalis* [G, Te, Co, E] *S. epidermidis* (MRSE) [O, Am, Cf, I, Le, G, Te, Co, E, Cl, Fu, R, Li]	*P. mirabilis* [Tg] *E. faecalis* [Co] ***C. albicans***
5	*E. faecalis* [G, S, Te, Co, E] *S. aureus* (MRSA) [O, Am, Cf, I, Le, M, E, Cl] *S. epidermidis* (MRSE) [O, Am, Cf, I, Le, M, G, Te, Co, E, Cl, Fo, R]	*E. coli* (3MRGN) [Am, P, Cf, Ct, Cz, Ci, Le, Co]	*P. mirabilis* [Tg] *E. faecalis* [Co]
6	*E. faecalis* [G, S, Te, Co, E] *S. aureus* (MRSA) [O, Am, Cf, I, Le, M, E, Cl] ***C. albicans***	*E. coli* (3MRGN) [Am, P, Cf, Ct, Cz, Ci, Le, Co] *E. faecalis* [Te, E] ***C. glabrata***	Negative
7	*Enterobacter cloacae* complex [Am, Fo]	*S. epidermidis* (MRSE) [O, Am, Cf, I, Le, G, Te, Co, E, Cl, Fu, R, Li] ***C. glabrata***	
8	*S. aureus* (MRSA) [O, Am, Cf, I, Le, M, E, Cl] *E. cloacae* complex [Am, Fo]	*S. epidermidis* (MRSE) [O, Le, G, Te, Co, E, Cl, Fu, R] *E. faecium* [Am, I] ***C. glabrata*** ***C. albicans***	
9	*E. cloacae* complex [Am, Fo]	*S. epidermidis* (MRSE) [O, Le, G, Te, Co, E, Cl, Fu, R] *E. faecium* [Am, I, Co] *S. haemolyticus* [O, Le, G, Te, Co, E, Cl, Fo]	
10	*S. epidermidis* (MRSE) [O, Am, Cf, I, Le, M, G, Te, Co, E, Cl, R, Fu, Li] ***C. albicans***	Negative	
11	*S. epidermidis* (MRSE) [O, Am, Cf, I, Le, M, G, Te, Co, E, Cl, R, Fu, Li]		
12	*S. epidermidis* (MRSE) [O, Am, Cf, I, Le, M, G, Te, Co, E, Cl, Fo] ***C. albicans*** [An]		
13	Negative		

The detected fungal pathogen is shown in bold. Resistance to antibiotics or fungicides are shown in square brackets. The following abbreviations were used: *Am* ampicillin, *An* anidulafungin, *Cf* cefotaxime, *Cz* ceftazidime, *Ce* cefuroxime, *Ci* ciprofloxacin, *Cl* clindamycin, *Co* cotrimoxazole, *E* erythromycin, *Fo* fosfomycin, *Fu* fusidic acid, *G* gentamicin, *I* imipenem, *Le* levofloxacin, *Li* linezolid, *M* moxifloxacin, *O* oxacillin, *P* piperacillin /tazobactam, *R* rifampicin, *S* streptomycin, *Te* tetracycline, *Tg* tigecycline

### Case 1

The first case refers to a morbidly obese, female patient—a feeding victim with a body mass index (BMI) of 52 kg/m^2^ (height: 170 cm and weight: 180 kg)—who has been suffering from arterial hypertension, hyperthyreosis and depression. Prior to the femoral part replacement (distal two thirds of the femur) in our clinic in May 2016, the 54-year-old patient had undergone a primary total knee arthroplasty (TKA) of the left knee in April 2014. A plate osteosynthesis after distal periprosthetic femur fracture in July 2015, and a re-osteosynthesis due to a renewed diaphyseal fracture in the middle of the femur with fracture of the plate in September 2015 were performed, each procedure in a different hospital. Due to poor bone quality and corresponding loss of bone tissue through multiple fractures and revision surgeries, only a femoral part replacement was ultimately considered as an attempt to preserve the limb, when the patient was hospitalized in May 2016 after another periprosthetic fracture. A routine biopsy was taken pre-operatively for microbiological testing with a negative result. However, soon after the replacement surgery, the patient returned to our clinic in June 2016 with a fulminant periprosthetic joint infection caused by methicillin-resistant *Staphylococcus aureus* (MRSA). Hip-disarticulation became necessary to control the infection. Hypoalbuminaemia despite of the adipositas, postoperative bleeding anemia and repetitive lows of electrolytes in the blood samples characterized the patient’s course. The postoperative course of the patient remained difficult due to persisting and hard to control soft tissue infection and osteomyelitis, and several revision operations were required. In each surgery, microbiological samples were taken and tested for bacteria and fungi using standardized protocols. Due to the changing microbiological findings, nine different long-term antibiotics had to be used for resistogram-compliant treatment, but the infection was still progressing. In the course of time, infection with *Candida albicans* became evident. The long-term application of caspofungin (initial dose 70 mg, treatment dose 50 mg per day), resistogram-compliant antibiotics and the fitting of a long-term permanent drain at the amputation stump finally led to a safe wound healing (Table 2). The patient was discharged free of pain and self-mobile in a wheelchair after 148 days of hospital stay. Medication with one oral antimycotic (fluconazole) and two antibiotics (linezolid and ciprofloxacin) continued for another four weeks after discharge. In prolonged seroma accumulation with increased secretory output, a permanent drain was necessary to prevent complications and subsequent operations. The drain was regularly exchanged during out-patient check-ups and was eventually removed when secretion dried up in May 2017, one year after admission to the hospital. Quality of life increased once an exo-prosthesis was fitted. In a follow-up visit in February 2019, the stump presented itself as healed and without any signs of infection. The patient has been painless since discharge and is satisfied with the result. While nine different antibiotics were administered for a total of 176 days the patient’s condition only improved after an antimycotic was included in the treatment regime (caspofungin for 29 days, fluconazole for 28 days).

**Table 2:** List of administered antibiotics and antimycotics in time course and applied days during the reported hospital stay

	Administered antibiotics and antimycotics in time course				
Applied days	Case 1	Applied days	Case 2	Applied days	Case 3
26 26	Clindamycin 600 mg (3 × 1) i.v Levofloxacin 500 mg (2 × 1) i.v	4	Levofloxacin 500 mg (2 × 1) i.v	4 41 16 24 28	Clindamycin 600 mg (3 × 1) i.v Cotrimoxazol 960 mg (2 × 1) i.v Linezolid 600 mg (2 × 1) i.v Unacid 3 g (3 × 1) i.v Caspofungin 70 mg (loading dose)/50 mg (1 × 1) i.v
30	Vancomycin (2 × 1), level-controlled i.v	29 6	Linezolid 600 mg (2 × 1) i.v Fosfomycin 5 g (3 × 1) i.v	30 30 28	Ciprofloxacin 400 mg (3 × 1) p.o Amoxicillin 1 g (3 × 1) p.o Fluconazole 200 mg (2 × 1) p.o
30	Rifampicin 600 mg (2 × 1) i.v				
19 5 14 95	Linezolid 600 mg (2 × 1) i.v Rifampicin 600 mg (2 × 1) i.v Levofloxacin 500 mg (2 × 1) i.v Caspofungin 70 mg (loading dose)/50 mg (1 × 1) i.v	17 10	Meropenem 1 g (3 × 1) i.v Unacid 3 g (3 × 1) i.v		
65	Tigecyclin 100 mg (1 × 1 loading dose), DDD 50 mg (2 × 1) i.v	25	Vancomycin (2 × 1), level-controlled i.v		
22	Fosfomycin 5 g (3 × 1) i.v	25	Fosfomycin 5 g (3 × 1) i.v		
		29	Caspofungin 70 mg (loading dose) /50 mg (1 × 1) i.v		
28 28 28	Ciprofloxacin 400 mg (3 × 1) p.o Linezolid 600 mg (2 × 1) p.o Fluconazole 200 mg (2 × 1) p.o				

Indication of the antibiotics used, duration of application in days and type of application intravenously (i.v.) or oral (p.o.). Medication p.o. is to be regarded as discharge medication. *DDD* defined daily dose

### Case 2

The second case corresponds to a female patient with a number of co-morbidities including diabetes mellitus, polyneuropathy in both feet, restless-leg syndrome, arterial hypertension, chronic nicotine abuse (> 30 pack years), overweight (BMI 28.08 kg/m^2^), hepatosplenomegalia and hepatic steatosis. The patient showed a complex medical history with regard to orthopaedic procedures. After a traffic accident with tibial plateau fracture in 2007 and posttraumatic gonarthrosis, a primary TKA of the right knee was performed in 2008. An early infection with *S. aureus* in 2009 required a two-stage revision of the primary TKA. In 2011 the amputation of the left big toe due to phlegmonia and osteomyelitis was carried out. A second two-stage revision of the TKA was necessary in 2014 after a periprosthetic joint reinfection with MRSA has occurred, resulting in the arthrodesis of the joint. Additionally, during this hospital stay the patient suffered a periprosthetic femoral fracture requiring a total femoral replacement. In December 2017, the 59-year-old patient presented at our hospital with a wound dehiscence and with spontaneous drainage of turbid secretion from her right knee. The initial emergency treatment comprised a wound revision with drainage inlay and microbiological and histological sampling of the knee and hip joint. *Enterococcus faecalis* was identified as cause of the periprosthetic infection in the microbiological analysis of the liquid biopsies from both, the hip and knee joints. Due to the seriousness of the infection, a hip disarticulation was indicated. Because of the deep-rooted and persistent infection, a vacuum assisted closure-therapy (VAC) was necessary for 12 days postoperatively and a spacer (gentamycin and vancomycin loaded polymethylmethacrylat (PMMA) cement) was implanted into the bone defect at the socket of the hip joint to allow local antibiotics release. Despite improving wound conditions, an infection of the amputation stump developed over the course of the hospital stay and another interval of vacuum therapy for four days was required. The postoperative course proved difficult with complications including anasarca due to cardiac insufficiency, hypokalemia and hypoalbuminemia. The prolonged progression of the infection was caused by a constantly changing bacterial spectrum displaying various resistances which was detected by microbiological analysis of tissue samples in the subsequent interventions. After some time, an opportunistic fungal infection with *C. albicans* and *Candida glabrata* was detected. In summary, *E. faecalis*, *Enterococcus faecium*, methicillin-resistant *Staphylococcus epidermidis* (MRSE), *C. albicans* and *C. glabrata* were found during the hospital stay and treated with seven different antibiotics according to the resistogram (Table 2). However, the infection persisted under sole antibiotic treatment. Only the additional treatment with caspofungin for 95 days resulted in significant improvement of soft tissue conditions. Thus, it was possible to discharge the patient after 78 days on our ward in April 2018. An oral antibiotic and antifungal discharge medication were not necessary. Outpatient appointments showed an increasing convalescence of the patient. In the latest outpatient check-up in February 2019, the patient reported to be free of pain and very satisfied with the results. The amputation stump showed no irritation and no signs of infection.

### Case 3

**Fig. 1:**
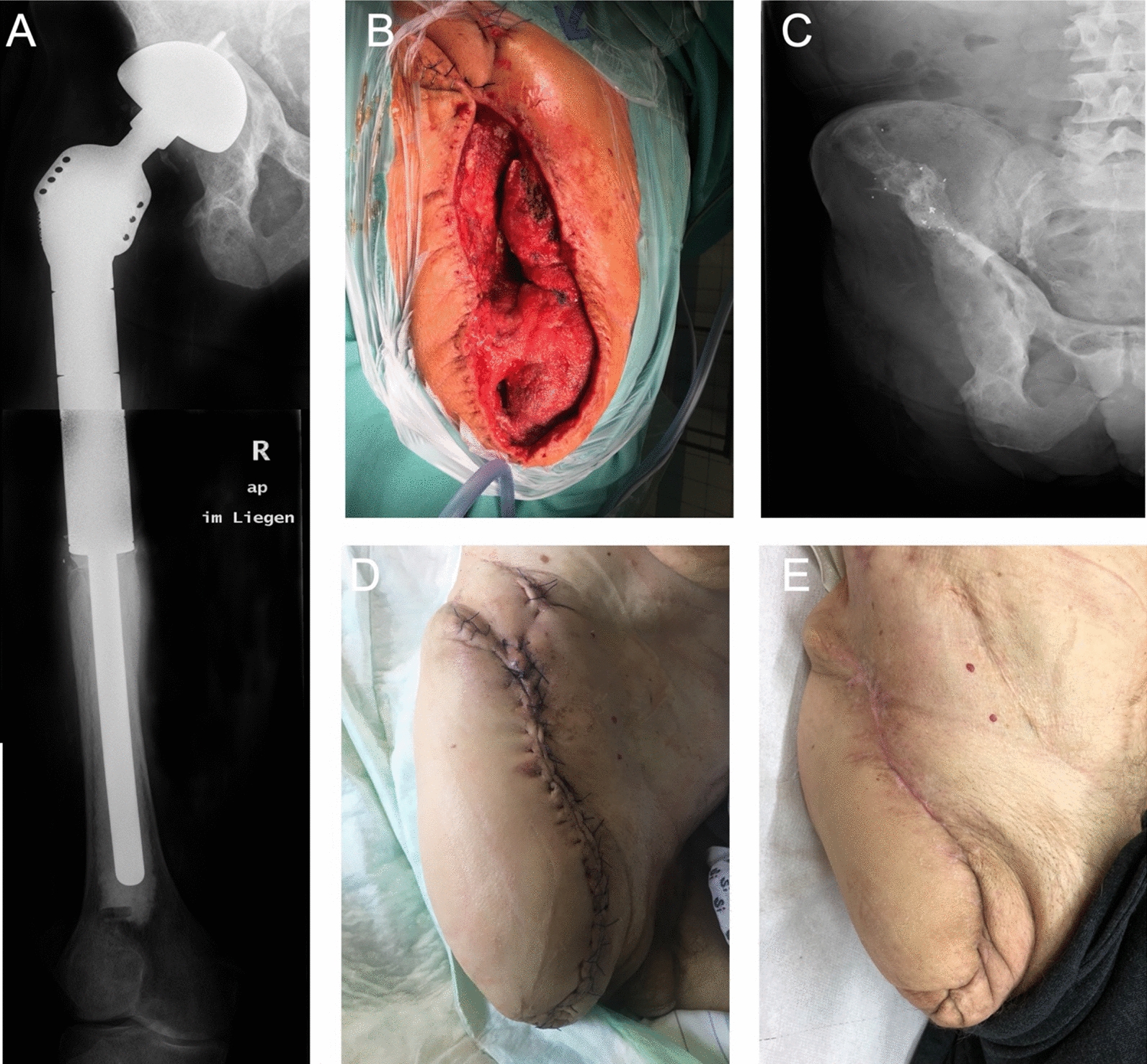
An example of the course of case 3. **A** shows the implanted endoprosthesis, **B** shows the final intraoperative result, **C** postoperative X-ray of the amputation stump, **D** wound healing during the course and **E** the healed stump 2 years postoperatively

The male patient in the third case report also underwent several orthopedic surgeries after the initial total hip arthroplasty. The primary hip arthroplasty was performed on the right side in 1996. This implant had to be removed in 2002 due to septic loosening. The reimplantation of a replacement was carried out in 2004. All procedures were done *alio loco*. The patient stated that at the time, he was coping well with the implantless condition and did not want a reimplantation. In 2007, the patient presented for the first time in our clinic, due to another periprosthetic infection. Then a one-step septic exchange of the total hip arthroplasty (THA) was performed. The one-stage exchange is one of the standard procedures for PJI, which is usually based on strict patient criteria and is performed in a selected patient population which meet certain requirements [10]. The high risk for periprosthetic infections was a consequence of the comorbidities of the patient. Apart from Type 2 diabetes mellitus, chronic hepatitis B, cardiac insufficiency and coronary heart disease, the patient suffered a congenital kidney failure with subsequent kidney transplantation in 1983. Graft failure in 1998 resulted in the need of dialysis and a second kidney transplantation in 2010. In particular, the immunosuppressive therapy after transplantation, which has been continuously administered until the present, increased the risk for infection. When the 53-year-old patient was admitted to our clinic, with fever and shivering in October 2017, a puncture of the right hip, confirmed a periprosthetic infection as the cause. Treatment was initiated with antibiotic therapy (see Table 2), removal of the infected implant and the implantation of an antibiotic loaded PMMA spacer. In March 2018, after consultation with the surgeons, the patient made the decision to have the lower limb amputated due to the further progression of inflammation and the resulting high functional insufficiency of the right lower limb with consecutive immobility and limited capability for personal hygiene. Post disarticulation, the soft-tissue infection and the osteomyeltitis of the acetabulum were difficult to treat. Besides VAC therapy and the use of an antibiotic-loaded PMMA-cement spacer, several surgical interventions were necessary. Upon detection of *E. faecalis* and *Proteus mirabilis,* therapy was adjusted. However due to the patient’s severe comorbidities, the health of the patient and the persisting infection of the amputation stump were hard to control. Despite the use of four different antibiotics, the final turning point was the start of antifungal therapy with caspofungin for 28 days after evidence of a *C. albicans* infection in the amputation stump. This, together with the last operative treatment, brought about the turnaround of the case. After 63 days of treatment, with necessary five follow-up interventions with debridement after the amputation procedure, it was possible to discharge the patient from the hospital. Wound healing was secure, the wound dry and in a non-irritant condition. Antifungal therapy was continued orally for four weeks after discharge with fluconazole. During regular check-ups, a stump swelling was observed, albeit without signs of inflammation. After the puncture of the swelling to drain the serous fluid during a short stationary stay, no further intervention was necessary. In March 2019 during a follow-up, the patient was in good condition with no evident pain and the stump was free of irritation. The clinical course of the patient is presented in Fig. 1.

## Discussion

This case series shows the worst cases of periprosthetic joint and bone infection. For infection detection, standardized tissue samples for microbiology were obtained intraoperatively at four sites in all cases according to Ellenrieder et al. [1]. Of these samples two were intramedullary tissue samples. Representative tissue was also sent for histological analysis of the periprosthetic membrane [1]. The treatment strategies ended with the loss of extremities. In summary, all patients were of younger age, had multiple revisions of the endoprosthesis, each had a large partial femoral replacement greater than 40% of the femoral length, gentamycin-loaded cement (in spacer and cement for endoprosthesis fixation), and a long anchoring distance of the used intramedullary stem. Multiple revision arthroplasties are very challenging, co-morbidities such as kidney transplantation, massive obesity, poorly controlled diabetes or massive nicotine abuse, like in our cases, complicate the treatment of difficult-to-handle bacterial infections. Another influencing factor is the presence of fungal infections. According to the literature they are considered rare [4, 11]. Infections with *C. albicans* and *C. glabrata* are the most common fungal pathogen which were also detected in our cases. [4]. Treatment algorithms for periprosthetic joint infections are established [12], but the recommendations for treatment of fungal infections of joints and bones are rare [13]. Intravenous application of antifungal agents is not well described for prosthesis infections [6]. So far, the most used antimycotic is fluconazole followed by amphotericin B [7]. Amphotericin B, fluconazole and echinocandins are recommend by 2016 IDSA-guidelines for Candida infections [13]. In the described cases of critically ill, septic patients, caspofungin was specifically chosen as the initial antifungal treatment. In candidemia, the use of an echinocandin as initial treatment is strongly recommended based on high-quality evidence [13]. Equally the German guidelines do not recommend the initial treatment with fluconazole anymore for critically ill patients with sepsis, but favour a echinocandin [14]. Contrary to fluconazole, some members of the echinocandins, such as caspofungin, can only be administered intravenously. However, the advantage of caspofungin is its biofilm penetrating effect [15, 16]. For fluconazole, this is only described for a significantly higher dosage with increasing side effects [9]. Treatment with caspofungin decreased a *C. glabrata* biofilm in an *in-vivo* mouse model while fluconazole treatment was ineffective [17]. The biofilm penetrating effect seems to be of special importance for the successful treatment of mixed bacterial and fungal infections. This makes caspofungin—an inhibitor of β-1,3-glucan synthesis in the fungal cell—an especially promising agent in the treatment of mixed fungal and bacterial infections. Indeed, treatment of mixed biofilms with a sub-inhibitory concentration of caspofungin, which did not suppress the growth of *Candida,* did not affect *S. aureus* directly. However, it restored the susceptibility of *S. aureus* towards vancomycin by diminishing the glucan synthesis and secretion [8]. Siala et al., demonstrated an adjuvant effect of caspofungin towards moxifloxacin activity in treating *S. aureus* biofilms [18]. The proposed mechanism is the destruction of the *S. aureus* biofilm matrix by inhibition of the bacterial N-acetylglucosamine transferase (IcaA), a homologue of the ß-1–3-glucan-synthetase, which is the fungal target of caspofungin. The decreased polymerization of the biofilm then would enable moxifloxacin to penetrate more deeply. In our cases PJI, represented mixed infections of *C. albicans* and bacteria that form mutually beneficial mixed biofilms with *Candida* species [8, 19–24]. It has been speculated that the resistance to antibiotics of certain bacteria in mixed biofilms is due to the secreted polysaccharides by Candida which form a barrier by coating the bacteria and thus physically prevent the interaction between the bacterial cell and the antibiotic [37, 39]. Therefore the biofilm-penetrating capability of caspofungin could be crucial for the success in treating PJI. Our findings in these three cases confirmed the effectiveness of caspofungin to treat mixed infections. The clinical turnaround, including the eradication of the bacterial pathogens, was only achieved after the initiation of treatment with caspofungin. This indicates that the effect of the antibiotics was restored.

## Conclusion

In these clinical cases with high but different risk profiles, a standardized treatment could not avoid the amputation of the affected limb with periprosthetic infection. Massive debridement and complete implant removal accompanied by antibiotic therapy were not successful. There was a delayed detection of candidiasis.

Only with the combination of further surgical intervention and the use of echinocandin-class antimycotics, the complex infection situation was finally controlled.

We therefore regard fungal infections not only as opportunistic infections, but as the crucial factor in the persistence of complex infections. The initial treatment with a biofilm penetrating fungicide in mixed fungal and bacterial infections might therefore be highly advantageous.

## Data Availability

The data were collected and evaluated within the Orthopaedic Clinic and Policlinic, University Rostock Medical Center, Rostock, Germany. The collected data obtained have been stored and are available at Orthopaedic Clinic and Policlinic.

## Abbreviations

PJI: Periprosthetic joint infection
BMI: Body mass index
TKA: Total knee arthroplasty
MRSA: Methicillin-resistant *Staphylococcus **aureus*
VAC: Vacuum assisted closure-therapy
PMMA: Polymethylmethacrylate
THA: Total hip arthroplasty
DDD: Defined daily dose

## References

[1] Ellenrieder M, Lenz R, Haenle M (2011). Two-stage revision of implant-associated infections after total hip and knee arthroplasty. GMS Krankenhhyg Interdiszip.

[2] Preobrazhensky PM, Bozhkova SA, Kazemirsky AV (2019). Functional outcome of two-stage reimplantation in patients with periprosthetic joint infection after primary total knee arthroplasty. Int Orthop.

[3] Arendrup MC (2013). Candida and candidaemia. Susceptibility and epidemiology. Dan Med J.

[4] Brown TS, Petis SM, Osmon DR (2018). Periprosthetic joint infection with fungal pathogens. J Arthroplasty.

[5] Kim J-K, Lee D-Y, Kang D-W (2018). Efficacy of antifungal-impregnated cement spacer against chronic fungal periprosthetic joint infections after total knee arthroplasty. Knee.

[6] Lee YR, Kim HJ, Lee EJ (2018). Prosthetic joint infections caused by Candida species: a systematic review and a case series. Mycopathologia.

[7] Cobo F, Rodríguez-Granger J, Sampedro A (2017). Candida prosthetic joint infection. a review of treatment methods. J Bone Jt Infect.

[8] Kong EF, Tsui C, Kucharíková S (2016). Commensal protection of Staphylococcus aureus against antimicrobials by Candida albicans biofilm matrix. MBio.

[9] Kuhn DM, George T, Chandra J (2002). Antifungal susceptibility of Candida biofilms: unique efficacy of amphotericin B lipid formulations and echinocandins. Antimicrob Agents Chemother.

[10] Rimke C, Enz A, Bail HJ (2020). Evaluation of the standard procedure for the treatment of periprosthetic joint infections (PJI) in Germany—results of a survey within the EndoCert initiative. BMC Musculoskelet Disord.

[11] Benito N, Franco M, Ribera A (2016). Time trends in the aetiology of prosthetic joint infections: a multicentre cohort study. Clin Microbiol Infect.

[12] Anemüller R, Belden K, Brause B, et al (2019) Hip and knee section, treatment, antimicrobials. In: Proceedings of international consensus on orthopedic infections. The Journal of Arthroplasty 34:S463–S475. Doi: 10.1016/j.arth.2018.09.032 10.1016/j.arth.2018.09.032 30348582

[13] Pappas PG, Kauffman CA, Andes DR (2016). Clinical Practice guideline for the management of Candidiasis: 2016 update by the infectious diseases society of America. Clin Infect Dis.

[14] Andreas H. Groll, Dieter Buchheidt, Werner Heinz, Romuald Bellmann, Oliver Cornely, Rainer Höhl, Martin Hönigl, Stefan Kluge, Oliver Kurzai, Cornelia Lass-Flörl, Thomas Lehrnbecher, Christoph Lichtenstern, Werner Mendling, Peter-Michael Rath, Volker Rickerts, Stefan Schwartz, Birgit Willinger, Markus Ruhnke (2020) S1 Leitlinie Diagnose und Therapie von Candida Infektionen: Gemeinsame Empfehlungen der Deutschsprachigen Mykologischen Gesellschaft (DMykG) und der Paul-Ehrlich-Gesellschaft für Chemotherapie (PEG) ICD 10: B37.-. Arbeitsgemeinschaft der Wissenschaftlichen Medizinischen Fachgesellschaften eV

[15] Høiby N, Bjarnsholt T, Moser C (2015). ESCMID guideline for the diagnosis and treatment of biofilm infections 2014. Clin Microbiol Infect.

[16] Bachmann SP, VandeWalle K, Ramage G (2002). In vitro activity of caspofungin against Candida albicans biofilms. Antimicrob Agents Chemother.

[17] Persyn A, Rogiers O, Brock M (2019). Monitoring of fluconazole and caspofungin activity against in vivo Candida glabrata biofilms by bioluminescence imaging. Antimicrob Agents Chemother.

[18] Siala W, Kucharíková S, Braem A (2016). The antifungal caspofungin increases fluoroquinolone activity against Staphylococcus aureus biofilms by inhibiting N-acetylglucosamine transferase. Nat Commun.

[19] Koo H, Andes DR, Krysan DJ (2018). Candida-streptococcal interactions in biofilm-associated oral diseases. PLoS Pathog.

[20] Fox EP, Cowley ES, Nobile CJ (2014). Anaerobic bacteria grow within Candida albicans biofilms and induce biofilm formation in suspension cultures. Curr Biol.

[21] Peters BM, Ovchinnikova ES, Krom BP (2012). Staphylococcus aureus adherence to Candida albicans hyphae is mediated by the hyphal adhesin Als3p. Microbiology (Reading).

[22] Morales DK, Hogan DA (2010). Candida albicans interactions with bacteria in the context of human health and disease. PLoS Pathog.

[23] Shirtliff ME, Peters BM, Jabra-Rizk MA (2009). Cross-kingdom interactions: Candida albicans and bacteria. FEMS Microbiol Lett.

[24] van Merode AEJ, Pothoven DC, van der Mei HC (2007). Surface charge influences enterococcal prevalence in mixed-species biofilms. J Appl Microbiol.

